# Supplementary figures and images for: Family-based whole-exome sequencing identifies novel loss-of-function mutations of FBN1 for Marfan syndrome
Source: PeerJ. 2018 Nov 13;6:e5927. doi: 10.7717/peerj.5927 (PMC6238762; doi:10.7717/peerj.5927)

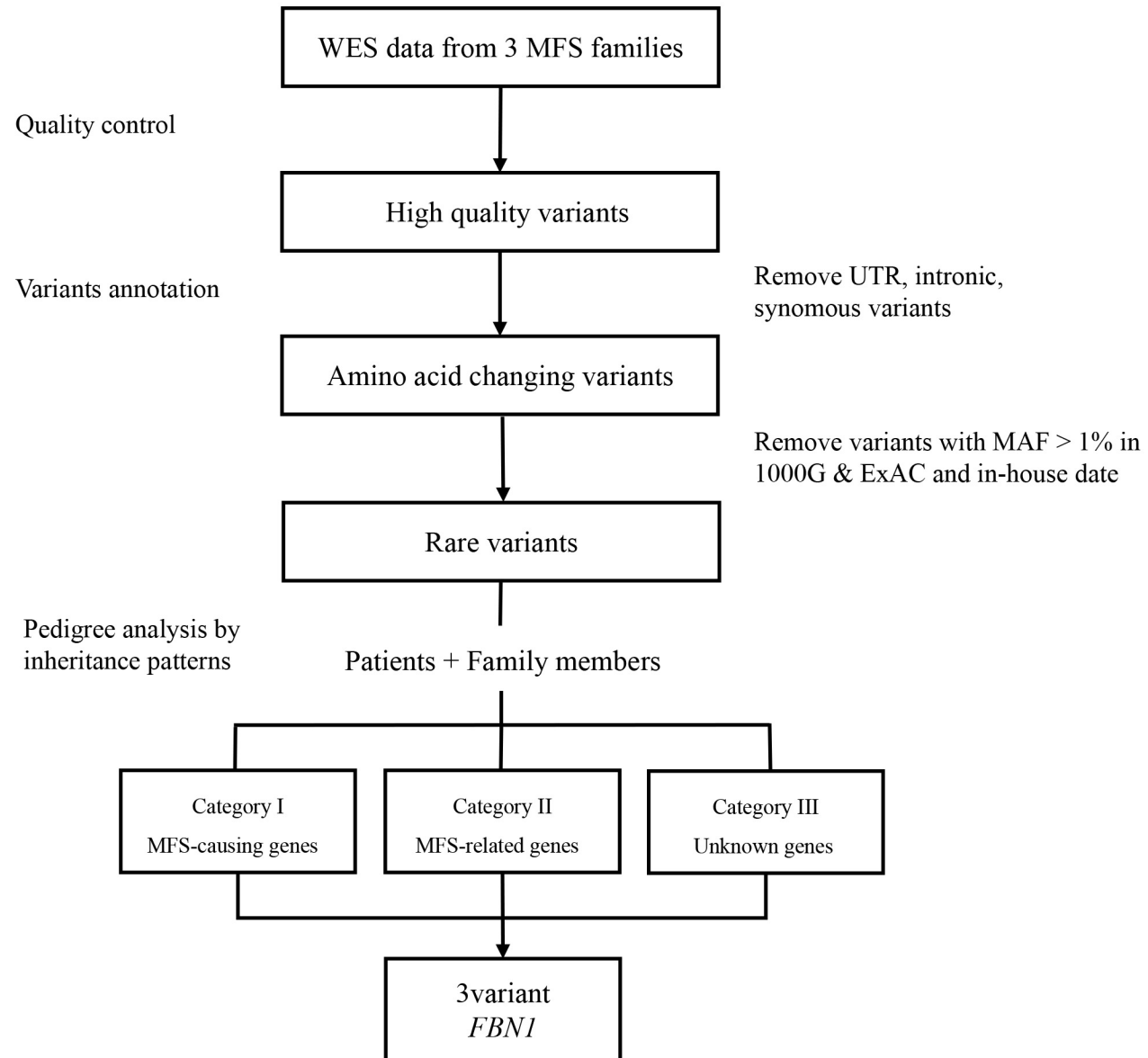

Supplement: Supplemental Information 1 — Abbreviations: MAF, minor allele frequency; UTR, untranslated region. [file peerj-06-5927-s001.pdf]
