# Supplementary material for: Family-based whole-exome sequencing identifies novel loss-of-function mutations of FBN1 for Marfan syndrome
Source: PeerJ. 2018 Nov 13;6:e5927. doi: 10.7717/peerj.5927 (PMC6238762; doi:10.7717/peerj.5927)
Supplement: Supplemental Information 2 — The positions of three LOF mutations in FBN1 are shown. The affected amino acid residues are conserved across multiple species. [file peerj-06-5927-s002.pdf]

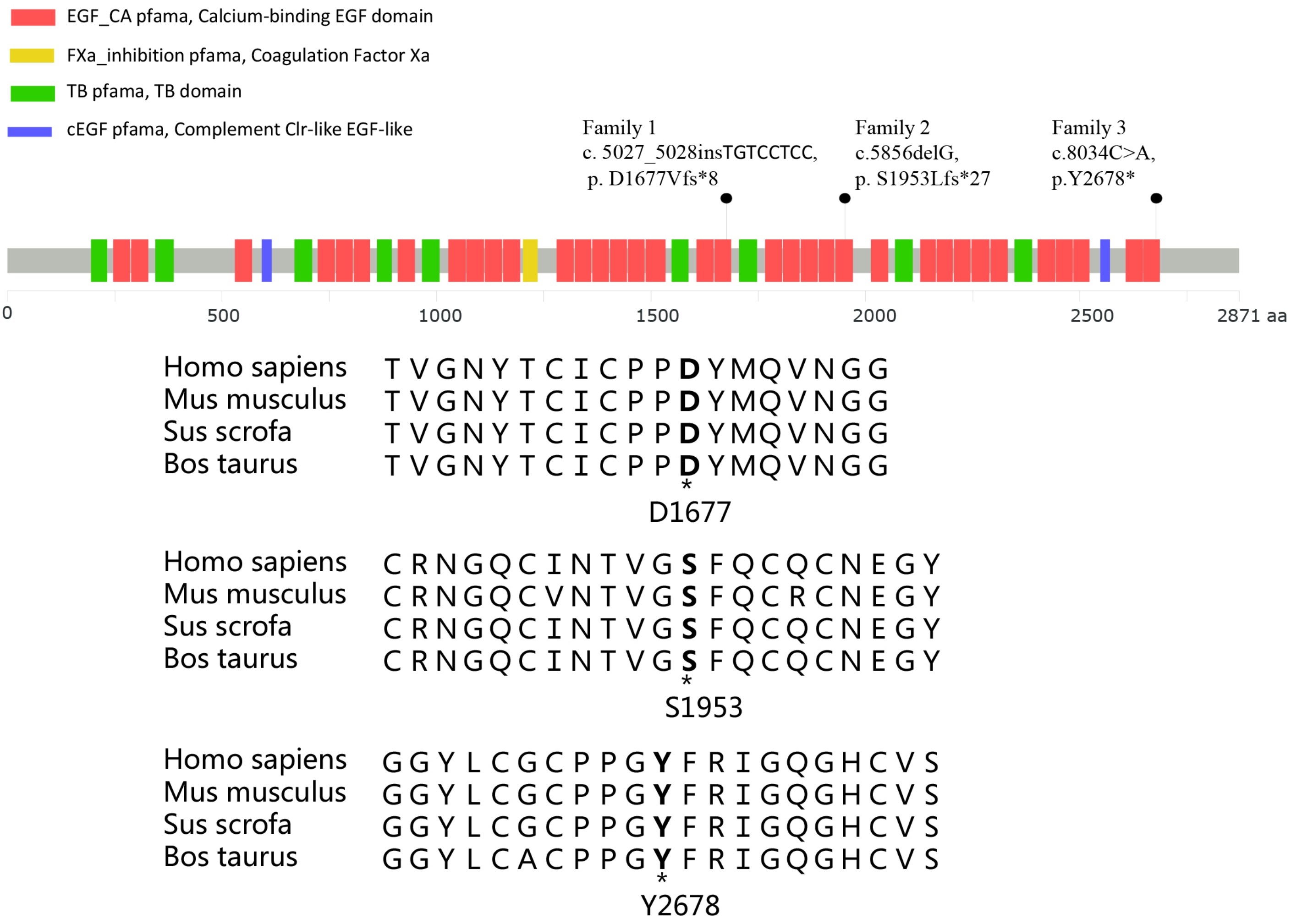
EGF_CA pfama, Calcium-binding EGF domain
FXa_inhibition pfama, Coagulation Factor Xa
TB pfama, TB domain
cEGF pfama, Complement Clr-like EGF-like
Family 1
c. 5027_5028insTGTCCTCC,
p. D1677Vfs*8
Family 2
c.5856delG,
p. S1953Lfs*27
Family 3
c.8034C>A,
p.Y2678*
0
500
1000
1500
2000
2500
2871 aa
Homo sapiens T V G N Y T C I C P P D Y M Q V N G G
Mus musculus T V G N Y T C I C P P D Y M Q V N G G
Sus scrofa T V G N Y T C I C P P D Y M Q V N G G
Bos taurus T V G N Y T C I C P P D Y M Q V N G G
*
D1677
Homo sapiens C R N G Q C I N T V G S F Q C Q C N E G Y
Mus musculus C R N G Q C V N T V G S F Q C R C N E G Y
Sus scrofa C R N G Q C I N T V G S F Q C Q C N E G Y
Bos taurus C R N G Q C I N T V G S F Q C Q C N E G Y
*
S1953
Homo sapiens G G Y L C G C P P G Y F R I G Q G H C V S
Mus musculus G G Y L C G C P P G Y F R I G Q G H C V S
Sus scrofa G G Y L C G C P P G Y F R I G Q G H C V S
Bos taurus G G Y L C A C P P G Y F R I G Q G H C V S
*
Y2678
