# Supplementary material for: Family-based whole-exome sequencing identifies novel loss-of-function mutations of FBN1 for Marfan syndrome
Source: PeerJ. 2018 Nov 13;6:e5927. doi: 10.7717/peerj.5927 (PMC6238762; doi:10.7717/peerj.5927)
Supplement: Supplemental Information 3 — #Category I: MFS-causing genes reported directly; Category II: 125 MFS-related genes from GeneCards; Category III: Unknown genes not reported previously. [file peerj-06-5927-s003.docx]

**Supplementary Table 1. Categories of MFS Pathogenic or Likely Pathogenic genes**

| Entrez Gene ID | Gene | Description | Category# |
| --- | --- | --- | --- |
| 2200 | *FBN1* | Fibrillin 1 | I |
| 7048 | *TGFBR2* | Transforming Growth Factor Beta Receptor 2 | I |
| 2201 | *FBN2* | Fibrillin 2 | I |
| 7046 | *TGFBR1* | Transforming Growth Factor Beta Receptor 1 | I |
| 4088 | *SMAD3* | SMAD Family Member 3 | I |
| 4638 | *MYLK* | Myosin Light Chain Kinase | I |
| 4629 | *MYH11* | Myosin Heavy Chain 11 | I |
| 59 | *ACTA2* | Actin, Alpha 2, Smooth Muscle, Aorta | I |
| 252842 | *AAT1* | Aortic Aneurysm, Familial Thoracic 1 | II |
| 176 | *ACAN* | Aggrecan | II |
| 1636 | *ACE* | Angiotensin I Converting Enzyme | II |
| 94 | *ACVRL1* | Activin A Receptor Like Type 1 | II |
| 11093 | *ADAMTS13* | ADAM Metallopeptidase With Thrombospondin Type 1 Motif 13 | II |
| 54507 | *ADAMTSL4* | ADAMTS Like 4 | II |
| 183 | *AGT* | Angiotensinogen | II |
| 185 | *AGTR1* | Angiotensin II Receptor Type 1 | II |
| 409 | *ARRB2* | Arrestin Beta 2 | II |
| 429 | *ASCL1* | Achaete-Scute Family BHLH Transcription Factor 1 | II |
| 632 | *BGLAP* | Bone Gamma-Carboxyglutamate Protein | II |
| 50947 | *BHD* | Beukes Familial Hip Dysplasia | II |
| 648 | *BMI1* | BMI1 Proto-Oncogene, Polycomb Ring Finger | II |
| 654 | *BMP6* | Bone Morphogenetic Protein 6 | II |
| 659 | *BMPR2* | Bone Morphogenetic Protein Receptor Type 2 | II |
| 796 | *CALCA* | Calcitonin Related Polypeptide Alpha | II |
| 821 | *CANX* | Calnexin | II |
| 824 | *CAPN2* | Calpain 2 | II |
| 875 | *CBS* | Cystathionine-Beta-Synthase | II |
| 948 | *CD36* | CD36 Molecule | II |
| 959 | *CD40LG* | CD40 Ligand | II |
| 1059 | *CENPB* | Centromere Protein B | II |
| 1060 | *CENPC* | Centromere Protein C | II |
| 91851 | *CHRDL1* | Chordin Like 1 | II |
| 1264 | *CNN1* | Calponin 1 | II |
| 80781 | *COL18A1* | Collagen Type XVIII Alpha 1 Chain | II |
| 1277 | *COL1A1* | Collagen Type I Alpha 1 Chain | II |
| 1278 | *COL1A2* | Collagen Type I Alpha 2 Chain | II |
| 1280 | *COL2A1* | Collagen Type II Alpha 1 Chain | II |
| 1290 | *COL5A2* | Collagen Type V Alpha 2 Chain | II |
| 1401 | *CRP* | C-Reactive Protein | II |
| 117154 | *DACH2* | Dachshund Family Transcription Factor 2 | II |
| 1634 | *DCN* | Decorin | II |
| 1908 | *EDN3* | Endothelin 3 | II |
| 1909 | *EDNRA* | Endothelin Receptor Type A | II |
| 1910 | *EDNRB* | Endothelin Receptor Type B | II |
| 1950 | *EGF* | Epidermal Growth Factor | II |
| 2006 | *ELN* | Elastin | II |
| 2099 | *ESR1* | Estrogen Receptor 1 | II |
| 2199 | *FBLN2* | Fibulin 2 | II |
| 84467 | *FBN3* | Fibrillin 3 | II |
| 201163 | *FLCN* | Folliculin | II |
| 2668 | *GDNF* | Glial Cell Derived Neurotrophic Factor | II |
| 2720 | *GLB1* | Galactosidase Beta 1 | II |
| 8349 | *HIST2H2BE* | Histone Cluster 2 H2B Family Member E | II |
| 3115 | *HLA-DPB1* | Major Histocompatibility Complex, Class II, DP Beta 1 | II |
| 3117 | *HLA-DQA1* | Major Histocompatibility Complex, Class II, DQ Alpha 1 | II |
| 3123 | *HLA-DRB1* | Major Histocompatibility Complex, Class II, DR Beta 1 | II |
| 3251 | *HPRT1* | Hypoxanthine Phosphoribosyltransferase 1 | II |
| 3329 | *HSPD1* | Heat Shock Protein Family D (Hsp60) Member 1 | II |
| 3381 | *IBSP* | Integrin Binding Sialoprotein | II |
| 3458 | *IFNG* | Interferon Gamma | II |
| 3586 | *IL10* | Interleukin 10 | II |
| 3552 | *IL1A* | Interleukin 1 Alpha | II |
| 3553 | *IL1B* | Interleukin 1 Beta | II |
| 3569 | *IL6* | Interleukin 6 | II |
| 3690 | *ITGB3* | Integrin Subunit Beta 3 | II |
| 23522 | *KAT6B* | Lysine Acetyltransferase 6B | II |
| 81930 | *KIF18A* | Kinesin Family Member 18A | II |
| 547 | *KIF1A* | Kinesin Family Member 1A | II |
| 26128 | *KIF1BP* | KIF1 Binding Protein | II |
| 3827 | *KNG1* | Kininogen 1 | II |
| 3912 | *LAMB1* | Laminin Subunit Beta 1 | II |
| 3913 | *LAMB2* | Laminin Subunit Beta 2 | II |
| 3952 | *LEP* | Leptin | II |
| 3956 | *LGALS1* | Galectin 1 | II |
| 4015 | *LOX* | Lysyl Oxidase | II |
| 4016 | *LOXL1* | Lysyl Oxidase Like 1 | II |
| 4035 | *LRP1* | LDL Receptor Related Protein 1 | II |
| 4041 | *LRP5* | LDL Receptor Related Protein 5 | II |
| 4053 | *LTBP2* | Latent Transforming Growth Factor Beta Binding Protein 2 | II |
| 4054 | *LTBP3* | Latent Transforming Growth Factor Beta Binding Protein 3 | II |
| 9968 | *MED12* | Mediator Complex Subunit 12 | II |
| 100507436 | *MICA* | MHC Class I Polypeptide-Related Sequence A | II |
| 4312 | *MMP1* | Matrix Metallopeptidase 1 | II |
| 4321 | *MMP12* | Matrix Metallopeptidase 12 | II |
| 4322 | *MMP13* | Matrix Metallopeptidase 13 | II |
| 4323 | *MMP14* | Matrix Metallopeptidase 14 | II |
| 4313 | *MMP2* | Matrix Metallopeptidase 2 | II |
| 4314 | *MMP3* | Matrix Metallopeptidase 3 | II |
| 4524 | *MTHFR* | Methylenetetrahydrofolate Reductase | II |
| 4548 | *MTR* | 5-Methyltetrahydrofolate-Homocysteine Methyltransferase | II |
| 4552 | *MTRR* | 5-Methyltetrahydrofolate-Homocysteine Methyltransferase Reductase | II |
| 4582 | *MUC1* | Mucin 1, Cell Surface Associated | II |
| 4724 | *NDUFS4* | NADH:Ubiquinone Oxidoreductase Subunit S4 | II |
| 4856 | *NOV* | Nephroblastoma Overexpressed | II |
| 10133 | *OPTN* | Optineurin | II |
| 64175 | *P3H1* | Prolyl 3-Hydroxylase 1 | II |
| 5034 | *P4HB* | Prolyl 4-Hydroxylase Subunit Beta | II |
| 5310 | *PKD1* | Polycystin 1, Transient Receptor Potential Channel Interacting | II |
| 10631 | *POSTN* | Periostin | II |
| 5468 | *PPARG* | Peroxisome Proliferator Activated Receptor Gamma | II |
| 5479 | *PPIB* | Peptidylprolyl Isomerase B | II |
| 5741 | *PTH* | Parathyroid Hormone | II |
| 5781 | *PTPN11* | Protein Tyrosine Phosphatase, Non-Receptor Type 11 | II |
| 5979 | *RET* | Ret Proto-Oncogene | II |
| 871 | *SERPINH1* | Serpin Family H Member 1 | II |
| 6441 | *SFTPD* | Surfactant Protein D | II |
| 285590 | *SH3PXD2B* | SH3 And PX Domains 2B | II |
| 6497 | *SKI* | SKI Proto-Oncogene | II |
| 6625 | *SNRNP70* | Small Nuclear Ribonucleoprotein U1 Subunit 70 | II |
| 6626 | *SNRPA* | Small Nuclear Ribonucleoprotein Polypeptide A | II |
| 6634 | *SNRPD3* | Small Nuclear Ribonucleoprotein D3 Polypeptide | II |
| 50964 | *SOST* | Sclerostin | II |
| 6696 | *SPP1* | Secreted Phosphoprotein 1 | II |
| 6741 | *SSB* | Sjogren Syndrome Antigen B | II |
| 3925 | *STMN1* | Stathmin 1 | II |
| 11075 | *STMN2* | Stathmin 2 | II |
| 7015 | *TERT* | Telomerase Reverse Transcriptase | II |
| 7040 | *TGFB1* | Transforming Growth Factor Beta 1 | II |
| 7042 | *TGFB2* | Transforming Growth Factor Beta 2 | II |
| 7043 | *TGFB3* | Transforming Growth Factor Beta 3 | II |
| 7049 | *TGFBR3* | Transforming Growth Factor Beta Receptor 3 | II |
| 79875 | *THSD4* | Thrombospondin Type 1 Domain Containing 4 | II |
| 7077 | *TIMP2* | TIMP Metallopeptidase Inhibitor 2 | II |
| 3371 | *TNC* | Tenascin C | II |
| 7124 | *TNF* | Tumor Necrosis Factor | II |
| 4982 | *TNFRSF11B* | TNF Receptor Superfamily Member 11b | II |
| 7133 | *TNFRSF1B* | TNF Receptor Superfamily Member 1B | II |
| 8600 | *TNFSF11* | Tumor Necrosis Factor Superfamily Member 11 | II |
| 8741 | *TNFSF13* | Tumor Necrosis Factor Superfamily Member 13 | II |
| 10673 | *TNFSF13B* | Tumor Necrosis Factor Superfamily Member 13b | II |
| 7138 | *TNNT1* | Troponin T1, Slow Skeletal Type | II |
| 137492 | *VPS37A* | VPS37A, ESCRT-I Subunit | II |
| 7481 | *WNT11* | Wnt Family Member 11 | II |

^#^Category I: MFS-causing genes reported directly; Category II: 125 MFS-related genes from GeneCards; Category III: Unknown genes not reported previously
