# Supplementary material for: Family-based whole-exome sequencing identifies novel loss-of-function mutations of FBN1 for Marfan syndrome
Source: PeerJ. 2018 Nov 13;6:e5927. doi: 10.7717/peerj.5927 (PMC6238762; doi:10.7717/peerj.5927)
Supplement: Supplemental Information 4 — * F: forward strand; R: reverse strand. [file peerj-06-5927-s004.docx]

**Supplementary Table 2. Primers for identified mutations**

| **family ID** | **Gene** | **Genetic mutations** | **Primer sequence** * | |
| --- | --- | --- | --- | --- |
| F1 | *FBN1* | c.5027_5028insTGTCCTCC, p.D1677Vfs*8 | F | CAGGCCATTCCAAAATGTGA |
|  |  |  | R | TTGTGAGCTCTCTTCCTCTTTG |
| F2 | *FBN1* | c.5856delG, p.S1953Lfs*27 | F | GCTGGGATTATGACATCTTTGG |
|  |  |  | R | TGCATGATTCCTTGAGTGGT |
| F3 | *FBN1* | c.8034C>A,p.Y2678* | F | TCAGTGGAGGATGCCAAGA |
|  |  |  | R | AAAGCATGGTTCTCCTCTGC |

* F: forward strand; R: reverse strand
