# Supplementary material for: Family-based whole-exome sequencing identifies novel loss-of-function mutations of FBN1 for Marfan syndrome
Source: PeerJ. 2018 Nov 13;6:e5927. doi: 10.7717/peerj.5927 (PMC6238762; doi:10.7717/peerj.5927)
Supplement: Supplemental Information 5 [file peerj-06-5927-s005.docx]

**Supplementary Table 3. Summary of original exome sequencing data**

| **Family ID** | **Member ID** | **Total_reads** | **Mapped_reads** | **Mapped_reads (%)** | **Duplicates** | **Duplicates (%)** | **Median_insert_size** | **GC (%)** |
| --- | --- | --- | --- | --- | --- | --- | --- | --- |
| F1 | I-1 | 53,050,802 | 52,934,860 | 99.78 | 2,951,376 | 5.56 | 191 | 45 |
| F1 | I-2 | 77,205,841 | 77,056,386 | 99.81 | 5,862,654 | 7.59 | 172 | 45 |
| F1 | II-1 | 103,867,981 | 103,666,909 | 99.81 | 8,422,650 | 8.11 | 175 | 45 |
| F2 | I-1 | 14,835,413 | 14,813,611 | 99.85 | 214,283 | 1.44 | 210 | 46 |
| F2 | I-3 | 23,547,611 | 23,518,992 | 99.88 | 451,288 | 1.92 | 186 | 46 |
| F2 | I-4 | 66,588,817 | 66,430,541 | 99.76 | 4,375,259 | 6.57 | 188 | 47 |
| F2 | I-5 | 50,190,109 | 50,074,186 | 99.77 | 3,008,468 | 5.99 | 188 | 47 |
| F2 | I-6 | 47,334,185 | 47,197,742 | 99.71 | 3,045,731 | 6.43 | 174 | 45 |
| F2 | II-1 | 71,226,839 | 71,087,013 | 99.80 | 4,246,336 | 5.96 | 183 | 45 |
| F2 | II-2 | 85,795,786 | 85,737,555 | 99.93 | 3,702,124 | 4.32 | 217 | 47 |
| F2 | II-3 | 65,552,917 | 65,403,562 | 99.77 | 4,476,058 | 6.83 | 187 | 47 |
| F2 | III-1 | 73,441,220 | 73,387,367 | 99.93 | 4,445,073 | 6.05 | 226 | 46 |
| F3 | I-1 | 92,078,624 | 91,964,691 | 99.88 | 4,550,684 | 4.94 | 219 | 46 |
| F3 | I-2 | 99,914,456 | 99,805,514 | 99.89 | 5,499,548 | 5.50 | 213 | 46 |
| F3 | I-3 | 99,072,434 | 98,952,949 | 99.88 | 4,627,309 | 4.67 | 209 | 47 |
| F3 | II-2 | 82,991,996 | 82,882,557 | 99.87 | 3,967,543 | 4.78 | 250 | 47 |
| F3 | II-3 | 68,592,044 | 68,377,603 | 99.69 | 2,296,996 | 3.35 | 216 | 47 |
| F3 | II-4 | 69,441,986 | 69,206,509 | 99.66 | 2,953,825 | 4.25 | 204 | 46 |
| F3 | III-1 | 74,962,852 | 74,733,994 | 99.69 | 2,534,412 | 3.38 | 202 | 46 |
